# Biomechanical and clinical evaluation of PSO, modified PSO and VCR treating OVCF kyphosis: a finite element analysis

**DOI:** 10.3389/fbioe.2024.1445806

**Published:** 2024-12-09

**Authors:** Junyu Li, Lizhi Xu, Haotian Wang, Yinhao Liu, Zhuoran Sun, Yongqiang Wang, Miao Yu, Weishi Li, Yan Zeng

**Affiliations:** ^1^ Department of Orthopaedics, Peking University Third Hospital, Beijing, China; ^2^ Engineering Research Center of Bone and Joint Precision Medicine, Beijing, China; ^3^ Beijing Key Laboratory of Spinal Disease Research, Beijing, China

**Keywords:** osteoporotic vertebral compression fracture, modified pedicle subtraction osteotomy, kyphosis correction, mechanical complications, perioperative parameters, finite element analysis

## Abstract

**Objective:**

To confirm the effect of surgery on spinal column biomechanics and to provide theoretical support for the advantages and disadvantages of different surgical methods and their clinical efficacy.

**Methods:**

33 continuous patients with no significant difference in risk factors related to the mechanical complications were enrolled in this retrospective study. Sagittal parameters were measured in the pre-, post-operative and following-up lateral radiograph of spine. An finite element (FE) model was created using CT scanning from a female volunteer with osteoporotic vertebral compression fracture (OVCF) with solely kyphosis. Pedicle subtraction osteotomy (PSO), vertebral column resection (VCR) and modified PSO(mPSO) for OVCF were simulated on FE model. Stress distribution and deformation of the FE model were measured.

**Results:**

Clinical - All differences in preoperative spinal sagittal parameters were not statistically significant. mPSO showed it is superior to PSO and VCR in multiple postoperative and following-up spinal sagittal parameters. The operation duration and intraoperative blood loss of mPSO are less than the other two. For postoperative mechanical complications, no statistically significant differences were observed. Biomechanical - Six operating conditions (flexion, extension, left/right bending, left/right twisting) for each post-operative FE model have been examined. In most conditions, the displacement of mPSO is similar to that of PSO, with both larger than that of VCR. All the maximum equivalent stress on the vertebral body is within the safe range. The stress is mainly distributed on the T10 vertebral body and the fixed vertebral body L2, while the stress of VCR is greater than that of mPSO and PSO. The intervertebral disc pressure is highest in VCR, followed by PSO, and lowest in mPSO under all conditions. The maximum pressure on the intervertebral discs is located between T10 and T11.

**Conclusion:**

The finite element analysis showed that mPSO has a similar spine stability to PSO, and possibly creates a better environment for bone-to-bone fusion and prevents adjacent segments degeneration. Combined with its less surgical risks, we believe that the modified pedicle subtraction osteotomy may be an appropriate strategy for indicated cases of OVCF.

## 1 Introduction

In the background of the aging population, osteoporotic vertebral compression fracture (OVCF) is one of the main complications of osteoporosis (Li et al., 2021). Patients with severe OVCF usually suffer from local kyphosis or even sagittal imbalance, which seriously affecting the patients’ quality of life and causing nerve damage and back pain (Jung et al., 2017). In the case of patients with severe OVCF with spinal deformity, open surgeries are required for treatments (Li et al., 2023).

For OVCF patients with severe kyphosis, osteotomies may be necessary to correct sagittal alignment, and it is effective for relieving neurological impairments due to spinal cord compression (Takahashi et al., 2016). Several osteotomy methods have been introduced into clinical practice. Smith-Petersen et al. (1969) first performed osteotomies (SPO) to correct lumbar kyphotic deformities in 1945. Later, pedicle subtraction osteotomy (PSO) achieves sagittal-plane correction by removing the posterior elements and making a vertebral body wedge (Gupta et al., 2020), with a correction of 25°–40° achieved (Bridwell et al., 2004). Larger correction up to 60° can be achieved by performing vertebral column resection (VCR), removing a whole vertebra and its upper and lower discs (Yang et al., 2016). Considering advanced age and severe osteoporosis in OVCF patients, there is high perioperative risk and high incidence of postoperative mechanical complications which leaves a clinical challenge to choose the optimal osteotomy method for orthopedic surgeons (Si et al., 2023).

Recently, some scholars have proposed to correct kyphosis by using a modified PSO osteotomy (Kim et al., 2020). This osteotomy method removes part of the injured vertebra, the upper half of pedicle, the adjacent damaged intervertebral disc, and achieves bone-to-bone fusion between the osteotomy surface and the adjacent vertebral body. This method can not only reduce surgical trauma, but also effectively correct kyphosis, accelerate fusion, and increase spinal stability. The modified PSO has shown good clinical outcomes and prognosis in treating some patients (Gao et al., 2015). However, its biomechanical rationale remains unclear.

Case series of various osteotomies have been reported to research on the best surgical approach for OVCF (Bridwell et al., 2004; Yang et al., 2016; Kim et al., 2020; Gao et al., 2015; Smith et al., 2008; Saifi et al., 2017). However, the above research results are only observations of clinical cases and have not been proven from the perspective of biomechanical mechanisms. Thus, we conduct this study, in which we compared the clinical outcomes of different osteotomy methods for OVCF and applied finite element analysis to evaluate the biomechanical features of a spine after several osteotomies, in order to verify the most ideal osteotomy method for OVCF kyphosis.

## 2 Methods

### 2.1 Clinical evaluation

We enrolled 33 continuous patients diagnosed with OVCF in total (11 for each) between 2016 and 2021 who underwent osteotomy in our institution. The surgical procedure was chosen by the surgeon based on the patient’s physical condition during the perioperative period and the surgeon’s mastery of the procedure. Three perioperative indicators, namely, the time of operation, intraoperative blood loss and hospitalization time, were collected in hospital information system. All the patients have received regular post-discharge follow-up. Data of the last follow-up was selected for evaluation of long term corrective effect. Spinal sagittal parameters were measured on patients’ lateral X-ray films by two experienced researchers independently at pre-, post-operative, and each follow-up time point. The radiological data were anonymous to researchers. If there is a significant difference in opinions between the two researchers, a third researcher will be asked to measure the parameters again; the two similar results will be adopted as the final result.

The measurement data are expressed as mean and standard deviation (x + s) and the enumeration data as the number of cases and percentage (x (%)). Measurement data is treated with the *t*-test and the One-Way ANOVA test and enumeration data is treated with the chi-square test. Data processing and analysis are done in SPSS Statistics 26.0 (IBM, United States).

### 2.2 Finite element analysis

#### 2.2.1 Construction of the thoracolumbar finite element model

A 71-year-old female volunteer who suffered from T12 OVCF underwent computed tomography scanning with slice thickness of 1 mm. A total of 272 computed tomography scan images were stored in Digital Imaging and Communications in Medicine (DICOM) format. The CT data were imported into the Mimics research 24.0 software (Materialise, Belgium) for 3-dimensional reconstruction. The optimization and materialization of the reconstructed vertebral body was done in Geomagic Studio 2013(Geomagic, United States). The intervertebral discs and the endplates were constructed in Solidworks 2016 (Dassault Systèmes, France). Abaqus 2016 software (Dassault Systèmes, France) was performed to define the material properties, set the boundary and loading conditions, mesh division, calculate conditions and accomplish FE analysis.

The finite element contains a total of 5 segments, namely, T10-L2, including vertebrae, intervertebral discs, endplates, and ligaments. The intervertebral disc was composed of a fibrous ring and a nucleus pulposus. Ligaments include the anterior longitudinal ligament, posterior longitudinal ligament, ligamentum flavum, interspinal ligament, supraspinal ligament, and transverse process ligament. The material properties of the model are shown in Table 1.

**TABLE 1:** The material properties of the finite element analysis model.

Structure	Elastic modulus (MPa)	Poisson ratio	Cross-sectional area	References
Cortical bone	12,000	0.3	-	Lavaste et al. (1992) Skalli et al. (1993) Shirazi-Adl et al. (1984)
Cancellous bone	100	0.2	-	Lavaste et al. (1992) Skalli et al. (1993)
Endplate	1,000	0.4	-	Lavaste et al. (1992)
Posterior element	3,500	0.25	-	Li et al. (2015)
Fibrous ring	4.2	0.45	-	Lavaste et al. (1992) Skalli et al. (1993)
Nucleus pulposus	1	0.49	-	Ding et al. (2020)
Cage, screw, and rod	110,000	0.3	-	Ambati et al. (2015)
anterior longitudinal ligament	7.8	0.3	63.7	Kim et al. (2012)
posterior longitudinal ligament	10	0.3	20.0	Kim et al. (2012)
ligamentum flavum	15	0.3	40.0	Kim et al. (2012)
interspinal ligament	10	0.3	40.0	Kim et al. (2012)
supraspinal ligament	10	0.3	30.0	Kim et al. (2012)
transverse process ligament	10	0.3	1.8	Kim et al. (2012)

#### 2.2.2 Construction of the postoperative models

A total of 3 postoperative models were constructed in this study, namely, PSO, VCR, and modified PSO (mPSO).

**FIGURE 1:**
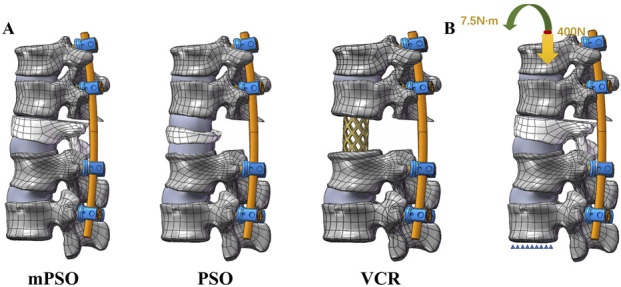
**(A)** Lateral views of postoperative finite element models of mPSO, PSO and VCR **(B)** Loading of the finite element model. The lower surface of the L2 vertebral body is fixed. A pressure of 400 N and a torque of 7.5 Nm are applied to the upper surface of the T10 vertebral body.

The PSO model was constructed by removing the T12 lemina, transverse processes, pedicle, and a wedge-shaped part of the vertebral body directly reaching the anterior cortical bone. The resection gap is closed along the cortical bone residue. The VCR model was constructed by completely removing the pathological vertebra and the adjoining upper and lower intervertebral discs. A titanium cage was placed to support the anterior column of the spine. The mPSO model was improved from the PSO model, removing part of the injured vertebra, the upper half of pedicle, and the intervertebral space above the pathological vertebra (Figure 1A).

The construction of the implants is based on the postoperative CT scan. The 3-D internal fixation model was constructed according to the insertion angles of the screws and the bending of the connecting rods. There are two sizes of the pedicle screws, 5.5*35 mm for T10-11 segments and 5.5*45 mm for L1-L2 segments. The diameter of the connecting rods is 5.5 mm. The screws and the rods are all made of titanium alloy (Ti_6_Al_4_ V).

#### 2.2.3 Contact relations, boundary conditions, and loading

Based on the physiological structure of the thoracolumbar spine, the vertebral body and the endplate, as well as the fibrous ring and the nucleus pulposus of the intervertebral disc, will not separate when given an external load. Therefore, the contact relations of the endplates and the vertebral bodies, the intervertebral discs and the endplates, and the fibrous ring and the nucleus pulposus of the intervertebral discs are set as Tie. The small joint and facet joint surface are in frictional contact, with a friction coefficient of 0.2. For the mPSO model, the T11 lower endplate and T12 upper surface are set to have frictionless contact. For the PSO model, the contact surface between the upper and lower parts of the T12 vertebral body is set to have frictionless contact. For the VCR model, the contact surfaces between the T11 lower endplate or the L1 upper endplate and the titanium cage are set to have frictionless contact. The pedicle screws are in binding contact with the vertebral body.

A pure moment of 7.5 Nm combined with a compressive load of 400.0 N, which simulates the weight of the upper body, was applied at the coupling node on the upper surface of the T11 vertebra to simulate flexion, extension, left–right lateral bending and left–right axial rotation movements. According to the right-hand rule, six motion conditions can be simulated by applying a pure torque of 7.5 Nm on the X, Y, and *Z*-axes. The degrees of freedom of all nodes on the L2 lower surface are set to be fully constrained as the boundary conditions (Figure 1B).

#### 2.2.4 Mesh convergence verification

Ayturk and Puttlitz (Ayturk and Puttlitz, 2011) have reported that, axial rotation is the most sensitive motion to mesh grid resolution, and it is considered that the mesh grid is convergent if the stress difference corresponding to different mesh sizes is within 5%. L1-L2 vertebral bodies are tested. Three different sizes of mesh grids are selected, namely, Mesh 1 (grid size:0.5; no. of nodes: 459,276; no. of units: 2435313), Mesh 2 (grid size:1; no. of nodes: 130,943; no. of units: 621,923) and Mesh 3 (grid size:2; no. of nodes: 29,381; no. of units: 153,175). All other conditions remain consistent. Loading and boundary conditions: An axial rotational torque of 7.5 N m are applied to the coupling point on the upper surface of L1. The lower surface of L2 is fully constrained.

## 3 Results

### 3.1 Clinical results

The results of clinical case analysis are presented in Table 2 **.**

**TABLE 2:** Sagittal parameters measurement, perioperative parameters, and post-operative complications.

		VCR	PSO	mPSO	*P*_*1*_	*P*_*2*_	*P*_*3*_
*Spinal sagittal parameters*							
LL	Pre-op	39.24 ± 21.87	36.53 ± 15.91	42.52 ± 15.54	0.759	0.699	0.393
	Post-op	26.70 ± 17.83	43.24 ± 17.80	29.03 ± 9.07	**0.042** ^*^	0.704	**0.029** ^*^
	LFU	25.00 ± 13.23	38.58 ± 21.60	22.70 ± 9.65	0.161	0.675	**0.039** ^*^
TLK	Pre-op	44.74 ± 18.91	43.1 ± 12.30	35.76 ± 12.86	0.88	0.208	0.154
	Post-op	13.73 ± 7.68	13.94 ± 12.05	10.43 ± 9.94	0.962	0.394	0.465
	LFU	15.16 ± 9.36	17.61 ± 12.46	15.43 ± 11.66	0.632	0.956	0.676
TK	Pre-op	27.47 ± 18.85	32.09 ± 17.59	41.58 ± 16.48	0.598	0.091	0.23
	Post-op	24.38 ± 13.93	30.57 ± 13.15	26.26 ± 9.96	0.361	0.734	0.415
	LFU	24.94 ± 4.96	22.34 ± 14.82	31.39 ± 12.21	0.717	0.291	0.217
TKmax	Pre-op	49.55 ± 21.09	55.59 ± 13.78	48.88 ± 12.43	0.436	0.928	0.245
	Post-op	18.98 ± 8.68	32.45 ± 10.36	21.31 ± 10.07	**0.004** ^**^	0.568	**0.019** ^*^
	LFU	20.34 ± 10.99	29.77 ± 13.44	22.76 ± 17.27	0.145	0.724	0.384
SVA	Pre-op	77.16 ± 74.73	52.14 ± 25.66	33.21 ± 32.76	0.385	0.099	0.193
	Post-op	34.17 ± 14.60	45.79 ± 20.05	19.88 ± 7.80	0.161	**0.010** ^*^	**0.001** ^**^
	LFU	21.39 ± 23.61	46.84 ± 25.22	35.39 ± 28.22	**0.049** ^*^	0.288	0.377
TPA	Pre-op	29.74 ± 9.18	36.43 ± 4.26	33.13 ± 10.83	0.869	0.617	0.726
	Post-op	29.74 ± 9.18	36.43 ± 4.26	21.82 ± 3.66	**0.049** ^*^	**0.015** ^*^	**<0.001** ^***^
	LFU	31.30 ± 13.20	28.78 ± 13.86	18.52 ± 6.60	0.782	0.053	**0.024** ^*^
*Perioperative parameters*							
Time of Operation		300.8 ± 30.9	301.4 ± 41.4	244.1 ± 63.0	0.487	**<0.001** ^***^	**0.006** ^**^
Intraoperative Blood Loss		1,236.3 ± 772.4	1,160.5 ± 650.4	790.0 ± 552.2	0.112	**0.016** ^*^	**0.033** ^*^
Hospitalization Time		12.0 ± 5.7	10.0 ± 2.0	10.5 ± 4.6	0.461	0.85	0.677
*Post-operative complications*							
		VCR(n = 11)	PSO(n = 11)	mPSO(n = 11)	*Significance*		
Complications		7 (63.6)	7 (63.6)	8 (72.7)	0.873		
Adjacent Segment Disease		2 (18.2)	0 (0)	2 (18.2)	0.32		
Distal Junction Problem		4 (36.4)	6 (54.5)	5 (45.5)	0.693		
Proximal Junction Problem		4 (36.4)	1 (9.1)	2 (18.2)	0.439		
Internal Fixation Problem		4 (36.4)	3 (27.3)	1 (9.1)	0.315		
Re-surgery		1 (9.1)	0 (0)	0 (0)	0.357		

LFU, last following-up; LL, lumber lordosis; TLK, thoracolumbar kyphosis; TK, thoracic kyphosis; TKmax, thoracic kyphosis maximum; SVA, sagittal vertical axis; TPA, T1 pelvic angle, Pre-op, pre-operative; Post-op, post-operative.

*P*_1_, *p*-value VCR, vs PSO; *P*_2_, *p*-value VCR, vs mPSO; *P*_3_, *p*-value PSO, vs mPSO.

^*^ *P* < 0.05^**^ *P* < 0.01^***^ *P* < 0.001.

All differences in preoperative spinal sagittal parameters were not statistically significant. Compared to PSO, mPSO showed a significant delince in postoperative lumbar lordosis (LL), maximal thoracic kyphosis (TKmax), sagittal vertical axis (SVA) and T1 pelvic angle (TPA), as well as in last following-up TPA. The difference in post-operative SVA and TPA values between mPSO and VCR is statistically significant.

In terms of perioperative indicators, the operation duration and intraoperative blood loss of mPSO are less than the other two surgical methods. The difference is statistically significant. For postoperative complications, no statistically significant difference between groups were observed.

### 3.2 Finite element analysis results

#### 3.2.1 Results of grid convergence testing

The number of units and nodes with different mesh grid sizes is described in methods Section 2.2.4, while Table 3 shows the stress percentage of the same position with different mesh grid sizes. According to the calculation results, the difference between the calculation results of Mesh 2 and Mesh 1 is within 5% except for the nucleus pulposus, and the difference between Mesh 3, Mesh 1, and Mesh 2 is relatively large. Therefore, the size of Mesh 2 can be considered convergent and can be used for subsequent calculations.

**TABLE 3:** Results of grid convergence verification showing the percentage difference of stress. It is used to determine the mesh that is suitable for analysis.

Portion	Mesh 1 and mesh 3	Mesh 1 and mesh 2	Mesh 2 and mesh 3
Cortical bone	1.21%	0.25%	1.46%
Cancellous bone	14.83%	4.14%	11.12%
Posterior structures	2.60%	1.01%	3.59%
Endplates	27.65%	1.94%	26.2%
Nucleus pulposus	33.67%	5.01%	29.75%
Fibrous ring	49.65%	4.96	47.02%

#### 3.2.2 Results of finite element analysis

**FIGURE 2:**
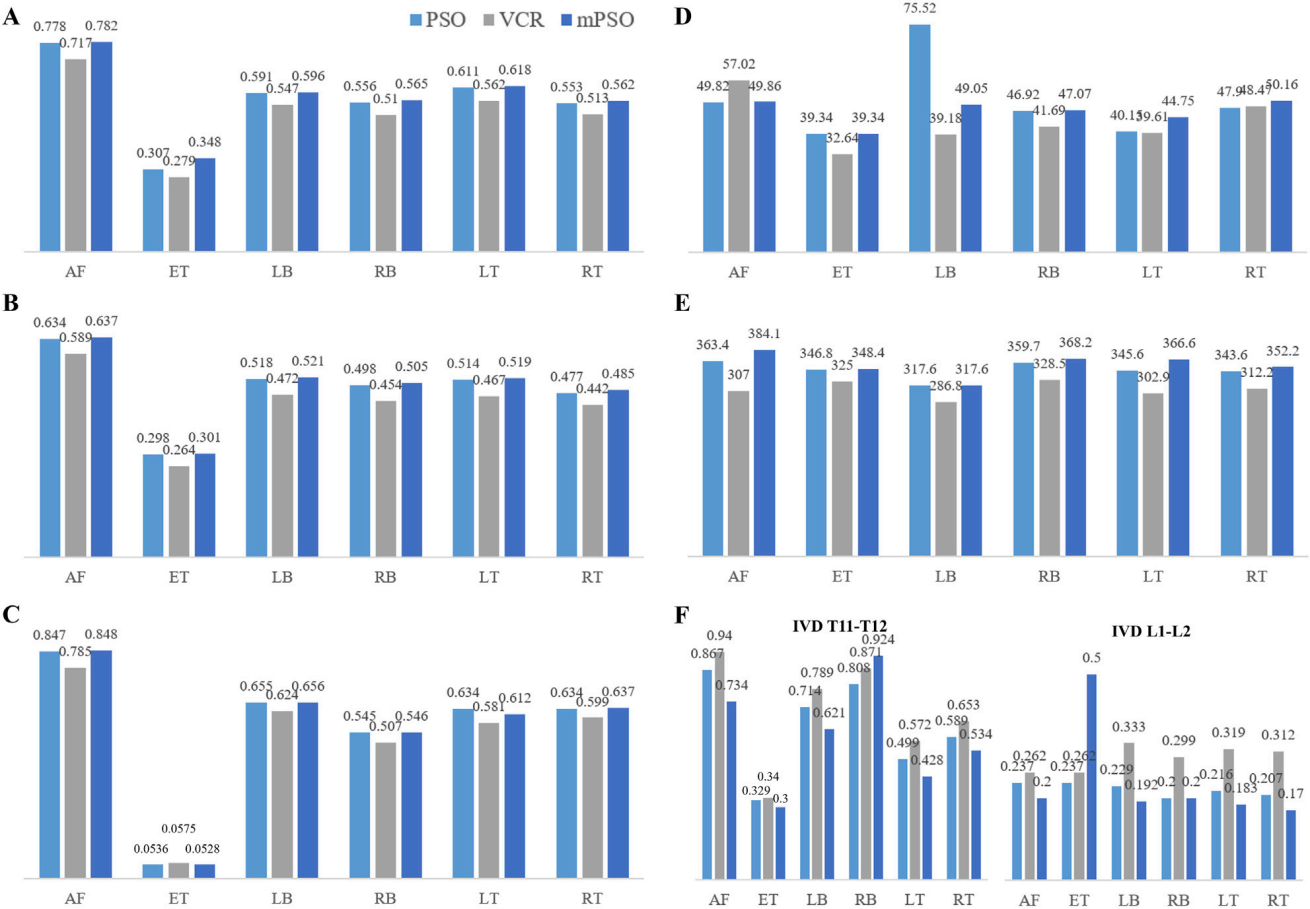
The results of finite element analysis showing the stability of the model **(A)** Displacement of the targeted vertebrae (mm) **(B)** Displacement of the internal fixation plant (mm) **(C)** Rotation of the targeted vertebrae (°) **(D)** von Mises stress (MPa) on the vertebral body **(E)** Maximal stress of the internal fixation (MPa) **(F)** Intradiscal Pressure (MPa). (AF: anteflextion; ET: extension; LB: left bending; RB: right bending; LT: left-handed twist; RT: right-handed twist).

The VCR model was observed a less displacement compared to the other two (Figure 2A). The displacement of internal fixation always exhibits the phenomenon of VCR model being the smallest and mPSO model being the largest (Figure 2B). The mPSO model performed slightly better than the PSO model in most of the conditions, proving its trustworthy stability. The VCR model showed less rotation under all working conditions except for extension (Figure 2C).

**FIGURE 3:**
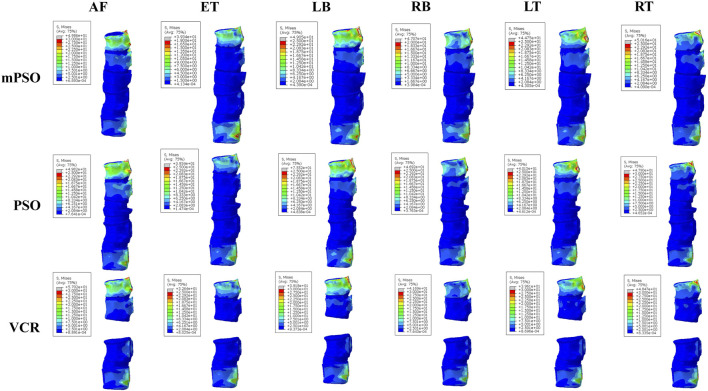
Heatmap showing von Mises stress (MPa) on the vertebral bodies of the postoperative models (AF: anteflextion; ET: extension; LB: left bending; RB: right bending; LT: left-handed twist; RT: right-handed twist). The stress is mostly concentrated on the T10 and L2 vertebrae, and all vertebrae are subjected to stress within a safe range.

A von Mises stress with significant difference was observed in the left bending condition of the PSO model, while other conditions showed not much difference. All stress are within the maximum stress range that the vertebral body can sustain (El-Rich et al., 2009) (Figures 2D, 3).

**FIGURE 4:**
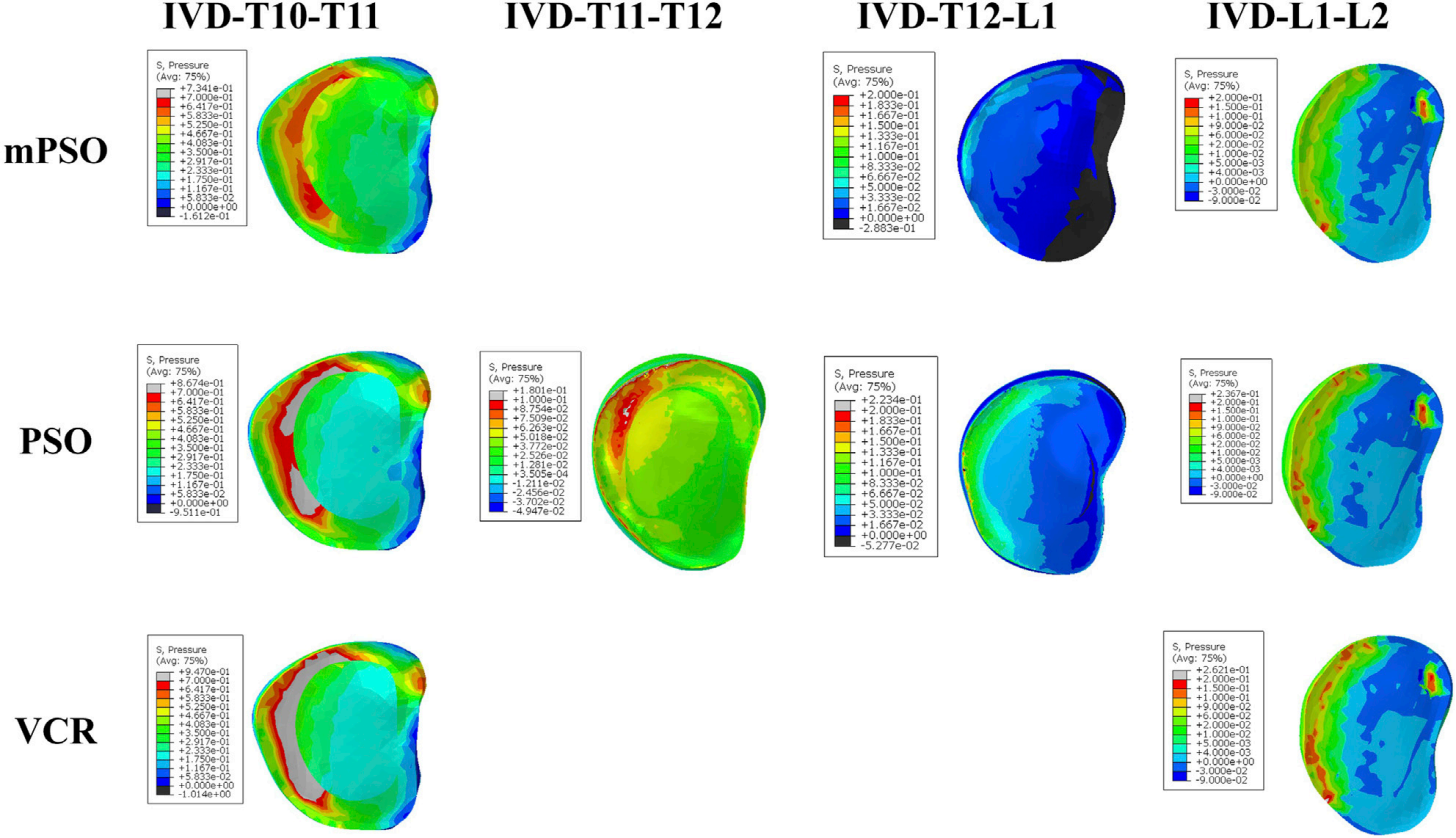
Heatmap showing the pressure on IVDs of the postoperative models under the working condition of anteflextion. IVDs in the VCR model bear the highest pressure. The pressure mainly concentrated in the front of IVDs.

Only the intervertebral discs reserved in all of the three models were counted, namely, T10-T11 and L1-L2 discs. The intradiscal pressure of the mPSO model was less than that of VCR except for in anteflextion working conditions. The overall trend is that VCR has the highest intradiscal pressure and mPSO has the lowest. All three models exhibit significant intradiscal pressure under flexion and lateral bending conditions, and the maximum stress region is located on the fibrous rings (Figure 2F). The maximum pressure is located in the IVD between T10 and T11, with the IVD of the VCR model of bears the highest pressure. For the mPSO model, there is tension on the upper surface of the IVD between T12 and L1, which is related to the motion state of the T12 vertebral body. In all six states of motion, the T12 vertebral body tends to move to the left and back, exerting a forward pulling force on the IVD between T12 and L1 (Figure 4).

**FIGURE 5:**
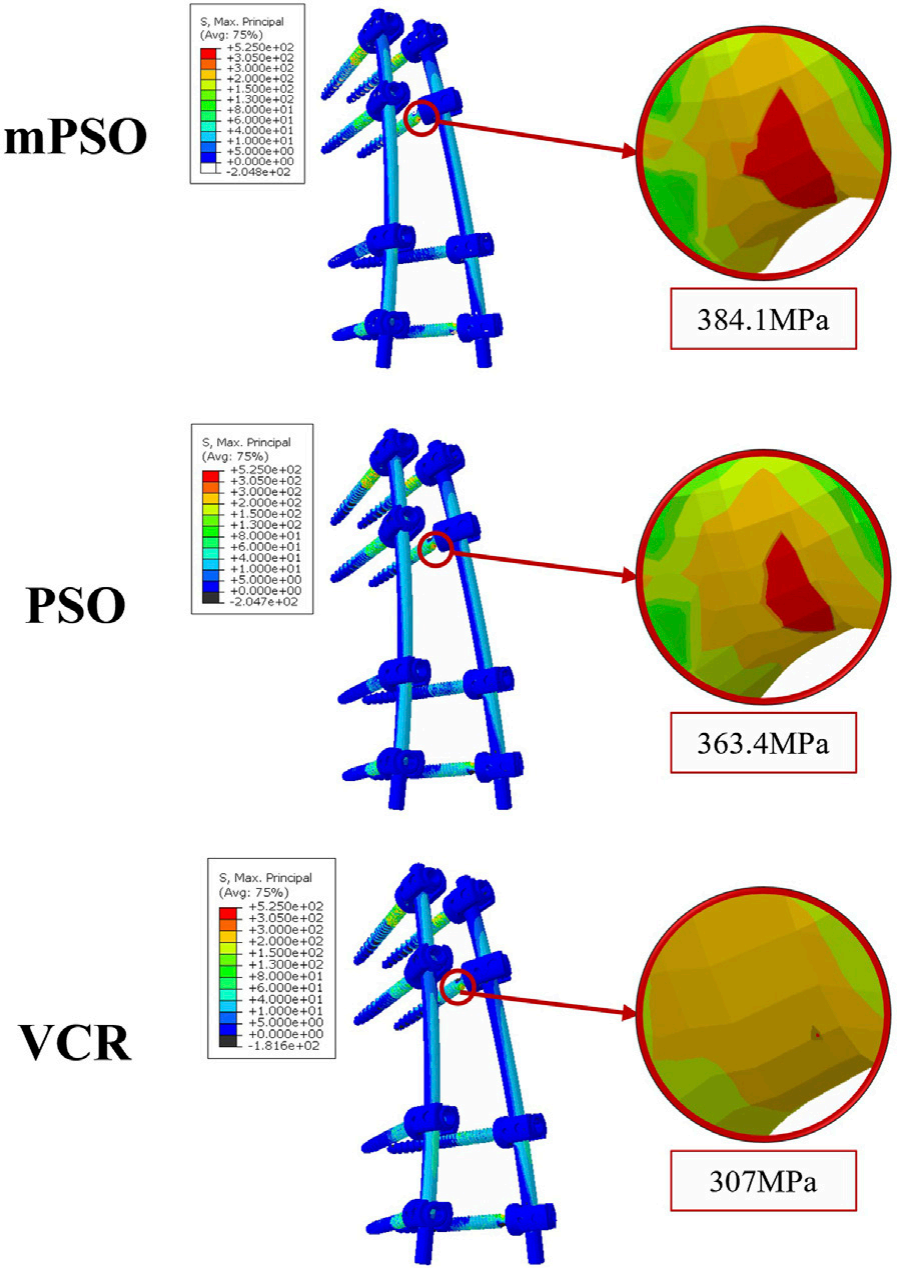
Heatmap showing the pressure on internal plantings of the postoperative models under the working condition of anteflextion. It shows the similar distribution of main stress in internal plants and there is stress concentration in the tail area of the screw.

The main stress distribution positions of the internal plants are the same, and there is stress concentration in the screw tail area. Under six different motion states, the main stress on the internal plant of the VCR procedure is the smallest, while the main stress on the internal plant of the mPSO procedure is slightly higher than that of the other two procedures (Figures 2E, 5).

## 4 Discussion

For OVCF with kyphosis, posterior osteotomy correction and internal fixation are the mainstream surgical methods. However, considering the patient’s advanced age and the presence of osteoporosis, there are often many comorbidities (Scheyerer et al., 2023). Therefore, how to choose the osteotomy method is currently a hot topic. Ponte osteotomy is less effective for severe deformity. Though possessing huge promise of both symptomatologic and radiological improvements, VCR is still a technically demanding procedure because of potentially significant blood loss, long surgical session and high risk of postoperative infection (Saifi et al., 2017; Lenke et al., 2010; Papadopoulos et al., 2015; Suk et al., 2002). Pedicle subtraction osteotomy (PSO) is one kind of three-column osteotomies, possessing the ability to achieve high grade of correction (Lau et al., 2014). Kim et al. (Kim et al., 2020) proposed a modified PSO by removing the diseased intervertebral disc on the basis of PSO. Cancellous bone were filled to the osteotomy surface of the target vertebra to create “bone-to-bone” contact. It has been also reported that mPSO has less intraoperative blood loss and shorter surgical time, while it has a favorable corrective result (Gao et al., 2015; Li et al., 2024). Therefore, we conducted this study, which compared the perioperative parameters of several osteotomy methods clinically, and used finite element analysis to compare the biomechanical characteristics.

In our clinical data review study, we found that mPSO has a good correction effect on OVCF kyphosis deformity, with better correction effect than PSO and not inferior to VCR. Especially the difference between SVA and TPA, the postoperative indicators of the mPSO group are close to normal, which is very gratifying for us. This may be due to the additional correction angle brought about by removing the diseased intervertebral disc. The mPSO group also showed better intraoperative safety compared to the other two groups. However, contrary to our expectation that mPSO has fewer complications, we did not observe significant differences in postoperative complications between mPSO and the other two surgical methods. This can be attributed to a smaller sample size, and on the other hand, may be due to the strict review of complications by us, which included some imaging manifestations of premonitory signs in the category of complications. It may also be due to the high risk of spinal surgery itself, which increases the likelihood of complications (Saifi et al., 2017).

Osteotomy alters the biomechanical situation of the spine. There is no consensus on which biomechanical changes caused by osteotomy are most beneficial for the correction of OVCF kyphosis. In order to explain the above results from a mechanistic perspective, we applied the finite element analysis method. Finite element analysis reconstructs the 3-D model of the spine based on the CT scanning results. It can simulate the forces on the spine under physiological conditions and calculate the displacement and deformation almost in real time. Besides, finite element models can be utilized repeatedly for surgery plan, research and education, allowing complicated surgical procedures to be simulated safely and efficiently. Hence, it has become a hot area of research.

In terms of global displacement, PSO has the largest displacement, whereas mPSO and VCR have smaller displacements than PSO, suggesting that mPSO has a superior stability. The surgical segment displacement (i.e., the displacement of the injured vertebra in the figure) of mPSO is the smallest, and that of PSO is the largest, indicating that the surgical segment mPSO has the optimal stability. VCR osteotomy achieves a good postoperative stability by placing a cage and applying compression, creating a rigid contact with the osteotomy surface. Meanwhile, modified PSO has advantages due to the maximized bone contact surface, leading to an early bone union, while keeping its efficacy of kyphosis correction (Kim et al., 2020).

In PSO, all the posterior elements and ligaments of the spine were resected by spine surgeons. The result is that the stability of the spine was maintained by only the bone to bone surface of the osteotomy site, the anterior intervertebral discs and the anterior longitudinal ligament. There was a high risk of postoperative rod fracture and spondylolisthesis due to poor local stability, especially in osteoporosis elderly patients (Bourghli et al., 2021). In VCR surgery, rigid fixation compensates for the lack of spinal support but the titanium cage subsidence was an another severe problem. In modified PSO, the lower facet joints were preserved, the upper 1/2 pedicle with damaged disc were removed to close intervertebral space and bone grafting were applying into it, thus to promote bone-to-bone fusion on osteotomy site and increase the postoperative stability maximally.

The stress on the adjacent endplates were the highest for VCR, followed by mPSO, and the lowest for PSO. For OVCF patients, the surgical segment is often located in the thoracolumbar region, where the spine transitions from the less mobile thoracic segment to the more mobile lumbar segment, which is prone to stress concentration. As a result, less adjacent endplate pressure was favorable. Besides, the patients usually suffer from osteoporosis, and the larger mechanical stress is more likely to cause mechanical complications. After VCR osteotomy, the relatively hard titanium cage is placed in the vertebral body, which causes the stress concentration on the osteotomy endplates, which is a problem worthy of attention. This often leads to vertebral fractures and titanium cage subsidence and other complications. PSO and mPSO have smaller stress on the endplates, and have significant advantages.

The stress is mainly distributed on the T10 and the fixed L2 vertebral bodies. In our study, the stress on the upper end of the VCR model is the largest, which probably leads to proximal junction kyphosis or failure. Although the maximum equivalent stress on the vertebral body is distributed within the safe range [155 MPa for cortical bones (El-Rich et al., 2009)] under all operating conditions of all models, fractures can more easily occur after long-term postoperative stress in the thoracolumbar junction of VCR model for lack of additional support from the rib cage. The open surgery of OVCF causes significant damage to the posterior structure of the spine, coupled with long segment fixation of the VCR procedure, which are all risk factors for proximal junction kyphosis (Bess et al., 2017; Kim et al., 2022).

Along with OVCF, there is often a certain degree of lesion in the intervertebral disc above the compressed segment which easily lead to the backpain due to continuous contact with each other. Therefore, reducing the stress on the proximal intervertebral disc during the osteotomy stage after surgery is an important consideration to avoid degeneration in the adjacent stage after surgery. The intervertebral disc stress after VCR surgery is the highest, and that after mPSO and PSO are smaller, indicating the potential advantages of mPSO in improving postoperative quality of life in OVCF patients. The influencing factors of adjacent segment degeneration include increased soft tissue stress and increased mobility (Virk et al., 2014). The preservation of the damaged intervertebral disc will cause conservative backpain. Residual intervertebral disc can also discourage bone fusion, and this unstable state may further lead to adjacent segment degeneration.

For VCR, the rigid fixation results in greater stress on adjacent intervertebral discs. For PSO, the inferior surface of the damaged upper disc is unavoidably exposed when the collapsed superior endplate is excised in the process of PSO, which may result in intervertebral disc deterioration in the future. Besides, referring the results of high postoperative displacement, PSO had higher risk of postoperative adjacent segment disease.

In summary, both VCR and PSO have a greater impact on adjacent segments. VCR has a greater impact on stress, while PSO has a greater impact on mobility. Overall, mPSO has a smaller impact on adjacent segments.

## 5 Conclusions

In clinical practice, SPO is less effective for OVCF with spinal deformity though it is commonly used. Applying VCR provides a good correction rate but the long surgical time, high surgical difficulty, high risk of intraoperative complications limit its application. PSO is more frequently applied because of its good corrective effects and relatively controllable trauma. However, insufficient removal of damaged intervertebral discs and compressed vertebral body may lead to the possibility of long-term complications. Therefore, mPSO is the operation of what we use in treatments with OVCF. Based on the changes of the sagittal parameters, we ensured that the effectiveness of mPSO is not inferior to VCR or PSO. And the safety of mPSO was reflected in our clinical practice. By fully removing the intervertebral disc and the injured part of compressed vertebral body, the targeted vertebra is able to directly contact with its upper vertebral body after osteotomy, promoting a bone-to-bone fusion and reducing the occurrence of mechanical complications.

## Data Availability

The data analyzed in this study is subject to the following licenses/restrictions: The data are not publicly available due to their containing information that could compromise the privacy of research participants. Requests to access these datasets should be directed to zengyanpku@163.com.
